# Sterically wrapping of multi-resonant fluorophores: an effective strategy to suppress concentration quenching and spectral broadening

**DOI:** 10.3389/fchem.2023.1198404

**Published:** 2023-05-05

**Authors:** Luo Xiaofeng, Zhang Dongdong, Duan Lian, Zhang Yuewei

**Affiliations:** ^1^ Key Lab of Organic Optoelectronics and Molecular Engineering of Ministry of Education, Department of Chemistry, Tsinghua University, Beijing, China; ^2^ Center for Flexible Electronics Technology, Tsinghua University, Beijing, China

**Keywords:** multiple resonance, narrowband emission, quenching resistance, wide concentration range, sterically wrapping strategy

## Abstract

Multiple resonance (MR) emitters are promising for the next-generation wide color gamut organic light-emitting diodes (OLEDs) with narrowband emissions; however, they still face intractable challenges such as concentration-induced emission quenching, exciton annihilation, and spectral broadening. In this concept, we focus on an advanced molecular design strategy called “sterically wrapping of MR fluorophores” to address the above issues. By isolating the MR emission core using bulky substituents, intermolecular interactions can be significantly suppressed to eliminate the formation of unfavorable species. Consequently, using the newly designed emitters, optimized MR-OLEDs can achieve high external quantum efficiencies of >40% while maintaining extremely small full width at half maxima (FWHMs) of <25 nm over a wide range of concentrations (1–20 wt%). This strategy may shed light on the design of efficient MR emitters, which provides more room for tuning the dopant concentrations under the premise of high-efficiencies and small FWHMs, accelerating the practical application of MR-OLEDs.

## Introduction

**FIGURE 1:**
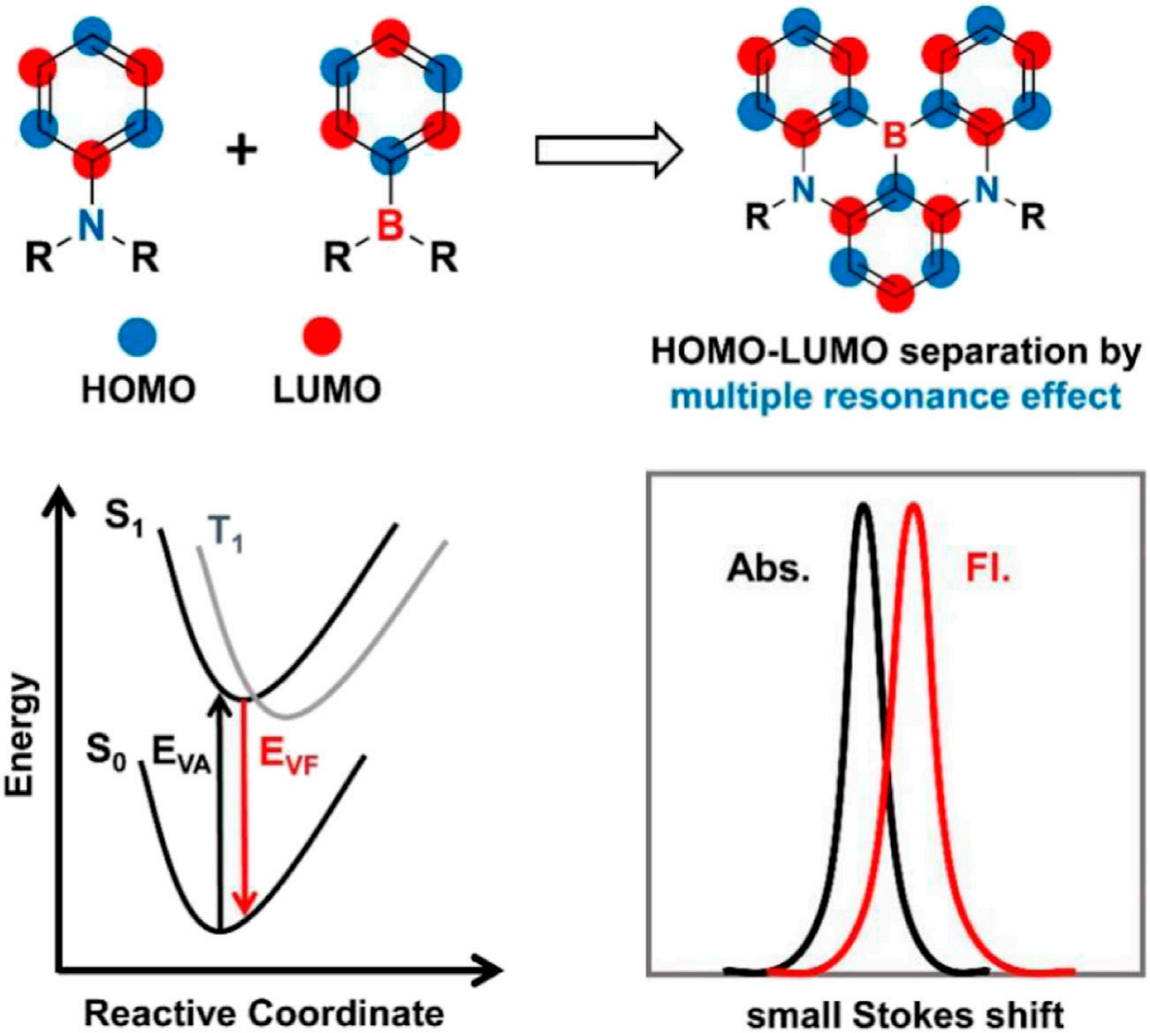
Design principle of MR emitters, where E_VA_ is vertical absorption and E_VF_ is vertical fluorescence.

Recently, the exploration of rigid boron and nitrogen-doped polycyclic aromatic hydrocarbons (B,N-PAHs) with multi-resonance thermally activated delayed fluorescence (MR-TADF) properties has attracted tremendous interest because of the great potential to provide extremely narrow bandwidth emission for next-generation wide color gamut displays (Hatakeyama et al., 2016; Kondo et al., 2019; Xu et al., 2020b; Kothavale and Lee, 2020; Madayanad Suresh et al., 2020; Suresh et al., 2020; Yang et al., 2020; Zhang et al., 2020; Zhang et al., 2021; Zou et al., 2022). These excellent properties result from the complementary resonance effects of electron-deficient (B) and electron-donating (N) atoms in the MR-TADF molecular backbone, which allow significant positioning of Frontier molecular orbitals (FMOs) on the atoms to minimize singlet-triplet energy gap (*ΔE*_ST_), binding/anti-binding properties and suppress vibrational coupling/relaxation (Figure 1). After nearly 7 years of development, MR-TADF emitters covering the entire visible region have been developed successively. Among them, *v*-DABNA (Kondo et al., 2019), BN-ICz-1 (Zhang et al., 2022a) and BBCz-R (Yang et al., 2020) represent the best performance of blue, green and red MR-OLEDs with FWHM/CIE color coordinates/EQE values of 18 nm/(0.12, 0.11)/34.4%, 23 nm/(0.22, 0.74)/30.5% and 26 nm/(0.67, 0.33)/22.0%, respectively.

Despite the booming development of materials with MR-TADF in terms of molecular structure, to the best of our knowledge, there is still a lack of research on supramolecular structures of such materials, which should undoubtedly be addressed for further applications. This is because the intrinsically planar structure of the MR-TADF emitter, which is prone to strong molecular aggregation, not only deteriorates the photoluminescence (PL) and electroluminescence (EL) efficiency, but also leads to broad excimer emission at high dopant concentrations (Stavrou et al., 2021). Therefore, most efficient MR-OLED devices are fabricated with low doping concentrations (∼1 wt%) to avoid the intense π-π interactions, which is obviously difficult to control during mass production evaporation. Indeed, since MR-TADF molecules have small irradiation shifts (*Δλ*_s_), low doping concentrations would result in incomplete energy transfer between host and guest, which not only broadens the EL spectrum (compared to the intrinsic PL spectrum in solution), but also reduces the device efficiency. The above situation is not significantly improved by the simple introduction of peripheral tert-butyl or other large protecting groups (Xu et al., 2020a). Therefore, an advanced molecular aggregation modulation strategy is urgently needed to solve the above concentration-induced problems related to emission quenching, exciton loss, spectral broadening, etc.

**FIGURE 2:**
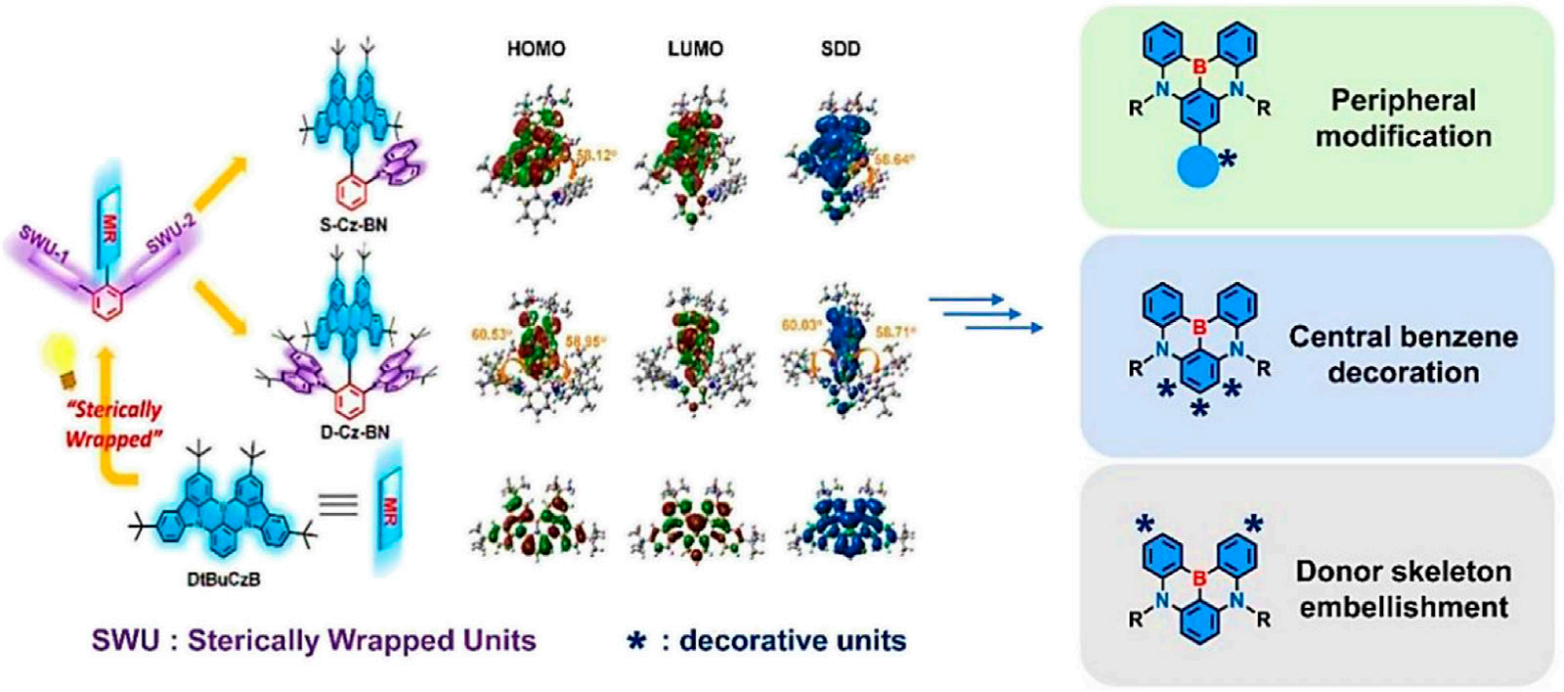
The molecular design strategy of “sterically wrapped MR dopants.” Adapted with permission (Zhang et al., 2022b). Copyright 2021, Wiley-VCH.

Inspired by conventional TADF materials, the rate constant of concentration quenching (*k*_CQ_) is a negative exponential function of the average intermolecular distance (*R*_0_) in the films (Lee et al., 2017), our group has recently proposed an advanced concept of “sterically wrapped MR dopants” with an MR-emitting core sandwiched between bulk substituents to solve the above issue (Zhang et al., 2022b) (Figure 2). In this circumstance, the distance between the MR-emitting core and neighboring molecules can be significantly increased, thus not only effectively suppressing the aggregation-induced emission quenching, but also solving the spectral broadening problem at the same time. As exemplified by S-Cz-BN and D-Cz-BN, where bulk substituents were introduced onto the peripheral phenyl of BCz-BN parent core at the para-B position, the HOMO-LUMO and lowest triplet state spin-density distributions (SDD) remained almost the same with that of BCz-BN. This particular form of FMO distribution not only imparts the typical MR properties of S-Cz-BN and D-Cz-BN, but also converts the introduced bulk groups into electronically inert moieties to effectively expand the intermolecular distance between MR-emitting cores, which should address the issue induced by dopant aggregation. Indeed, in contrast to the BCz-BN devices where remarkably enlarged FWHMs (from 29 to 36 nm) and significantly reduced *EQE*_max_ (from 27.5% to 18.1%) can be observed with increasing dopant concentrations, both the target sterically wrapped MR dyes could afford small FWHMs and high EQEs over a wide range of dopant concentrations (1–20 wt%). For S-Cz-BN and D-Cz-BN devices, the bilaterally shielded D-Cz-BN exhibited significantly higher *EQE*_max_s (36.3%–37.2%) and smaller unchanged FWHMs (24 nm) within the same concentration interval (1–20 wt%), superior to S-Cz-BN-based devices (FWHMs: from 26 to 30 nm; *EQE*_max_s: from 30.5% to 28.8%). This strategy elucidates the design of more efficient MR emitters, which provides more room for tuning the MR dopant concentration in the prerequisite of narrowband emission with high efficiency, thus attracting much attention from researchers. For a deeper understanding of this strategy, we have provided a brief summary of the progress in this concept. The selected MR-TADF materials were divided into the following three groups according to the connection between the site-resistant groups and the MR cores: peripheral-, central benzene ring-, and donor skeleton wrapping.

### Peripheral -wrapped MR emitters

**FIGURE 3:**
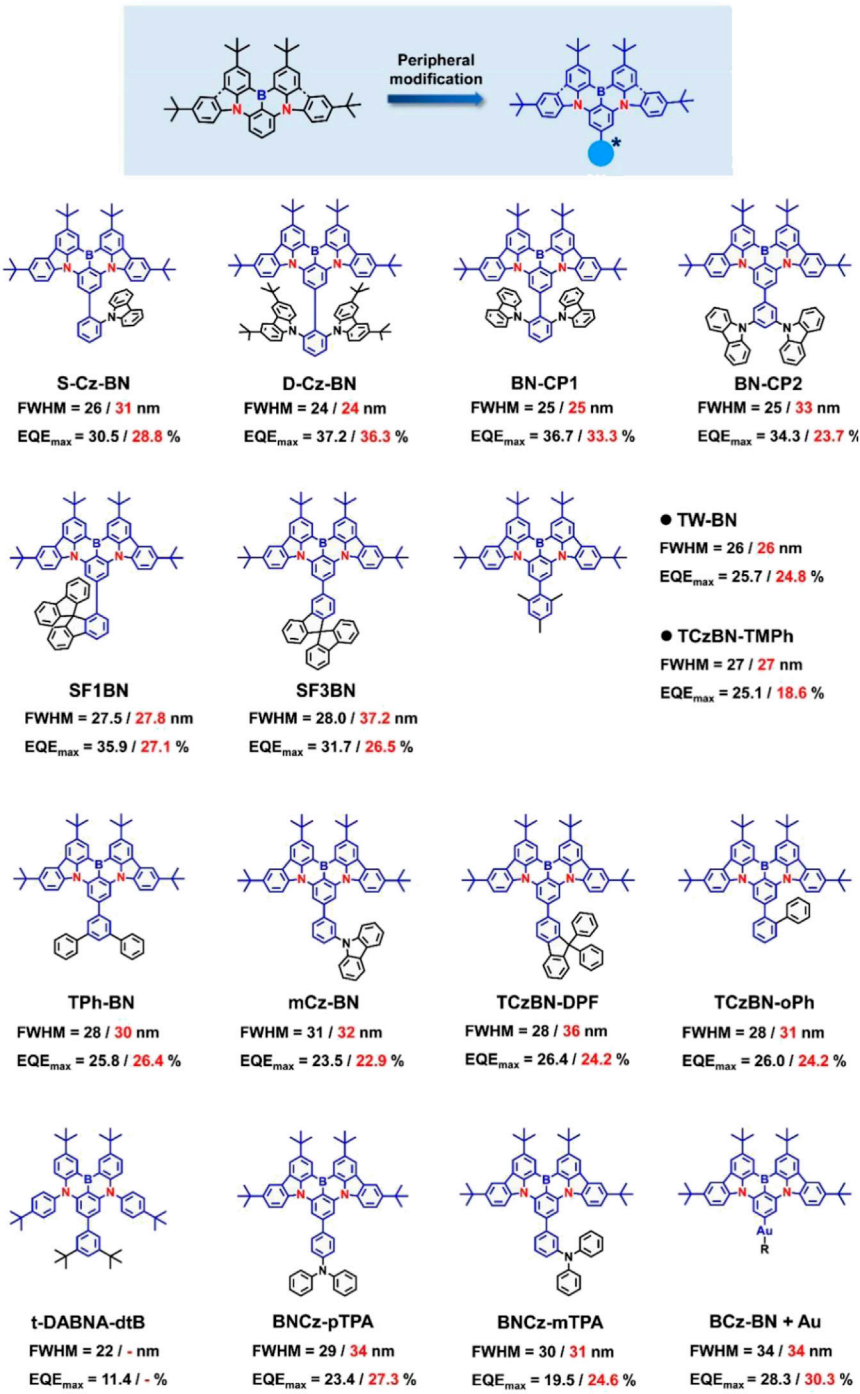
Chemical structures and device performances of peripherally wrapped MR emitters, where the values represent the FWHMs and EQE_max_s of the OLED devices at low (left) and high (right) doping concentrations, respectively.

Yang et al. (2020) reported a very similar wrapping MR-TADF emitter, BN-CP1, during the same period and compared its luminescence properties with those of the less-steric isomer, BN-CP2 (Jiang et al., 2022) (Figure 3). Among them, BN-CP1 with its unique three-dimensional geometry and steric effect is more favorable for suppressing chromophore interactions, which is verified by its superior photophysical properties in heavily doped films than BN-CP2. As a result, BN-CP1-based OLEDs show remarkable doping-insensitivity with *EQE*_max_s as high as 33.3%–40.0% and FWHM values maintained at 25 nm over the doping concentration range of 1 wt% to 30 wt%. Based on the similar concept, Jiang et al. (2022) introduced spiro-9,9′-bifluorene (SBF) as a monosubstituted steric hindrance group attached to the BCz-BN core to construct two MR isomers named SF1BN and SF3BN (Qu et al., 2022). The larger 3D bulky substituents and relatively rigid structures can help to hinder chromophore interactions and avoid additional vibrations, leading to high PLQYs and narrowband emissions. Benefiting from the more rigid structure and less vibrational relaxation, SF1BN based OLEDs exhibited higher *EQE*_max_s of 27.1%–35.9% and smaller FWHM values of ∼27 nm within a doping ratio of 2–15 wt%.

To simplify the molecular structure, Lu et al. reported a simple wrapping strategy by introducing peripheral phenyl derivatives as steric hindrance groups via single-bond linkages, which is also able to weaken the bimolecular interactions to obtain narrowband emissions and high *PLQY*s (Liu F. et al., 2022). Owing to the steric effect of the molecular edges, all MR-OLEDs exhibit small FWMH values of ≤32 nm and high *EQE*s of ≥22.9% with concentrations ranging from 1-3 wt%. Subsequently, by comparing MR-TADF emitters with the same core and different steric substituents, Zhang et al. demonstrated that the color purity and efficiency reduction in MR-OLEDs at high doping concentrations arise from the formation of excimer and triplet-state-related quenching between adjacent molecules, respectively (Huang et al., 2022). By selecting the appropriate steric substituent, high EQE_max_s of 26.0%–24.2% and invariably small FWHMs of 28–31 nm can be achieved simultaneously in TCzBN-oPh devices (1–20 wt%). Lee et al. (2017) developed a blue emitter called DABNA-dtB with a di-tert-butylphenyl (dtB) substituent attached to t-DABNA (Park et al., 2022). The introduction of dtB as a steric blocking group suppressed the intermolecular interactions and concentration quenching effects, so that the DABNA-dtB films showed extremely high PLQYs of ≥96% and an identical small FWHM of 22 nm in the doping concentration range of 3–7 wt%. The corresponding TTF-OLED achieved a high *EQE* of 11.4% and an extremely long lifetime with LT_95_ > 208 h at an initial luminance of 1,000 cd m^−2^.

Among other examples of peripheral-wrapped MR molecules, Huo et al. developed two efficient MR emitters, BNCz-pTPA and BNCz-mTPA, exhibiting aggregation-induced emission enhancement (AIEE) properties by attaching triphenylamine (TPA) onto BCz-BN cores (Chen et al., 2023). In this work, researchers pointed out that the introduction of electron-rich TPA not only suppressed the ACQ owing to its large twisted steric structure but also modulated the CT (charge-transfer) character in high-level triplet excited states for faster RISC while retained the LE (locally-excited) character of *S*_1_ for narrowband emission. Accordingly, both emitters showed rather high *PLQY*s ≥ 90% and small FWHMs of 22 nm (in toluene). Especially in doped films, the *PLQY*s of the two emitters increased continuously with increasing doping concentration, reaching a maximum of 98% at 5 wt% (BNCz-pTPA) and 10 wt% (BNCz-m-TPA), respectively. OLEDs based on the above AIEE-MR emitters afforded high EQE_max_s up to 27.3% with small efficiency roll-offs, which were much better than that of BCz-BN ones. Unanimously, Che et al. and Yang et al. reported a novel panel of interesting Au(I) MR-TADF emitters featuring BN(O)-based emitters covalently attached to Au-NHC patterns via Au-Caryl bond (Cai et al., 2022; Wang et al., 2023). Optically transparent (>350 nm) and sterically bulky NHC-assisted ligands were chosen here to expand the intermolecular distance between MR-emitting cores and to improve the stability of Au emitters. Thus, even at high doping level (20 wt%), an almost constant small FWHM of 37 nm could also be obtained, indicating the negligible π-π stacking interactions in these wrapping MR emitters. Notably, ultra-pure green OLEDs doped with (BzIPr)AuBN exhibit maximum *EQE*s > 30.3% and small FWHMs of ∼34 nm, as well as extremely low efficiency roll-off of 0.8% and an excellent operation stability (LT60) of more than 1,200 h at an initial luminance of 1,000 cd/m^2^.

### Central benzene-wrapped MR emitters

**FIGURE 4:**
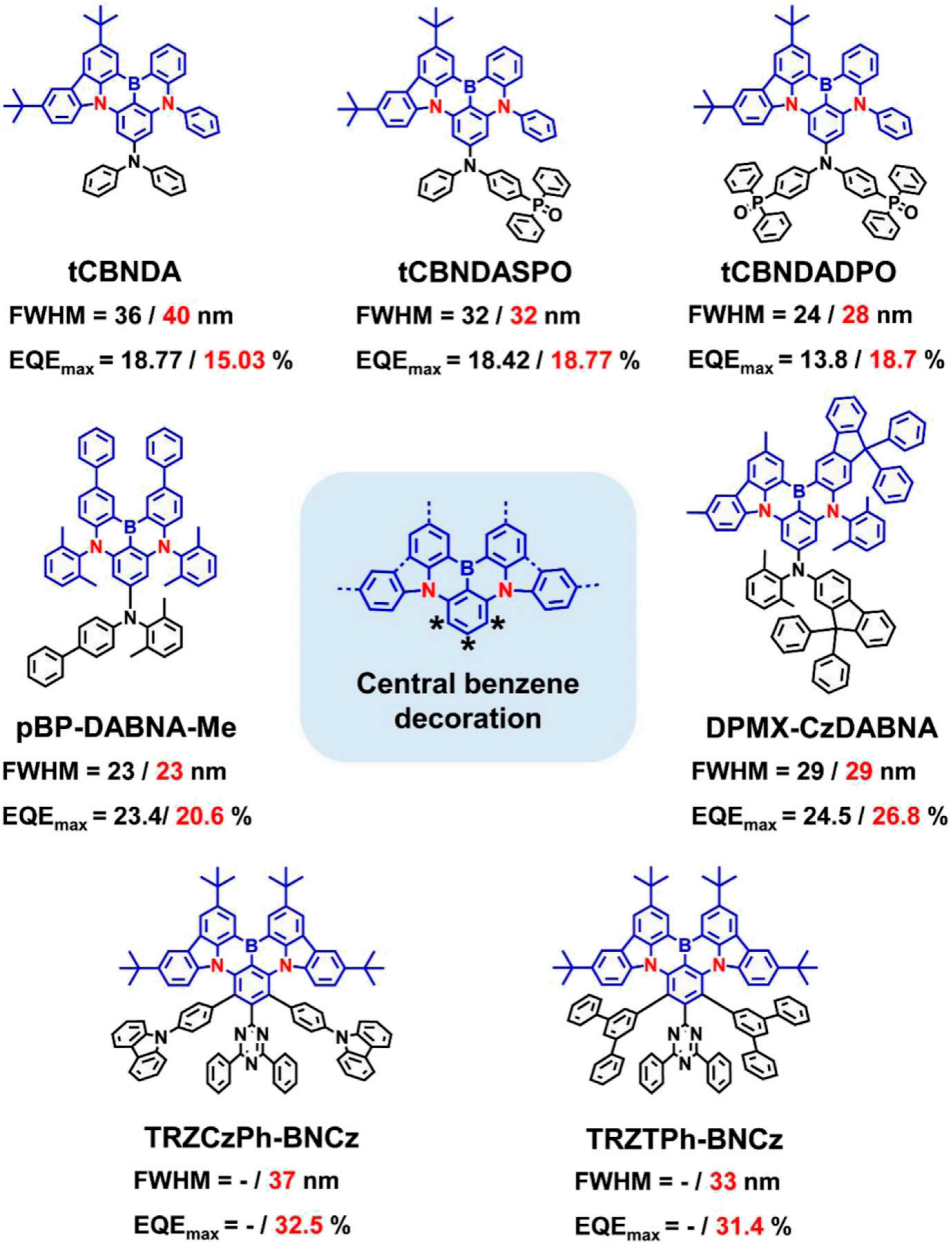
Chemical structures and device performances of central-benzene-decorative MR emitters, where the values represent the FWHMs and EQE_max_s of the OLED devices at low (left) and high (right) doping concentrations, respectively.

The three reactive sites on the central benzene ring of the MR core can also be well used for the construction of the wrapping structure. For example, Xu et al. proposed an “ambipolar self-host” strategy that integrated a molecular segment functioning as a bipolar host into the MR framework (Bian et al., 2022a; Bian et al., 2022b) (Figure 4). The introduction of DADPO (donor-acceptor type phosphine oxide) could effectively inhibit intermolecular interactions without affecting additional charge transfer or vibrational constituents to excited states. Thus, even at a high doping concentration of 30 wt%, tCBNDADPO showed state-of-the-art *PLQY* and *EQE*_max_ values of 99% and 30%, respectively, while maintaining a narrowband blue emission with an FWHM of ∼28 nm.

Through a simple synthesis process with the introduction of meta-xylene and meta-phenyl groups to the MR core, Kim et al. developed a blue MR emitter, mBP-DABNA-Me, with suppressed intermolecular interactions and isomer formation (Cheon et al., 2022). At a relatively high doping concentration (5 wt%), mBP-DABNA-Me was able to show an efficient narrowband emission with a peak at 467 nm, a small FWHM of 28 nm, and a high *PLQY* of 97%. Notably, as the doping rate increased from 5 to 20 wt%, the corresponding OLED devices exhibited superior performance with *EQE*s of 23.4%–20.6% and FWHMs of ∼28 nm. Soon afterward, Zhang et al. reported a similar wrapping blue MR-TADF molecule named DPMX-CzDABNA (Hu et al., 2022). In accordance with the previous results, the introduction of peripheral 9,9-diphenyl-9H-fluorene (DPF) and 2,6-dimethylphenyl contributed to the suppression of interchromophore stacking of emitters, which enabled the DPMX-CzDABNA-based OLEDs to achieve high *EQE*_max_s of 26.8%–24.5% with an identical small FWHM of 29 nm over a wide range of dopant concentrations (5–20 wt%).

Based on the concept of “space-confined donor-acceptor (SCDA),” You et al. developed two pure green MR dyes by attaching the bulk SCDA units onto the BCz-BN core, which not only helps to suppress spectral broadening and exciton quenching, but also is able to promote the multichannel RISC process by forming intermediate triplet states (Liu Y. et al., 2022). Thus, TRZCzPh-BNCz and TRZTPh-BNCz exhibited good photoluminescence performances with *PLQY*s > 83.7%, FWHMs ∼37 nm, and *k*_RISC_s > 0.75 × 10^6^ s^−1^ at doping ratios of 1–50 wt%. The optimized OLED was able to show a small FWHM of 37 nm and a high *EQE*_max_ of 32.5% with mitigated efficiency roll-off.

### Donor skeleton-wrapped MR emitters

**FIGURE 5:**
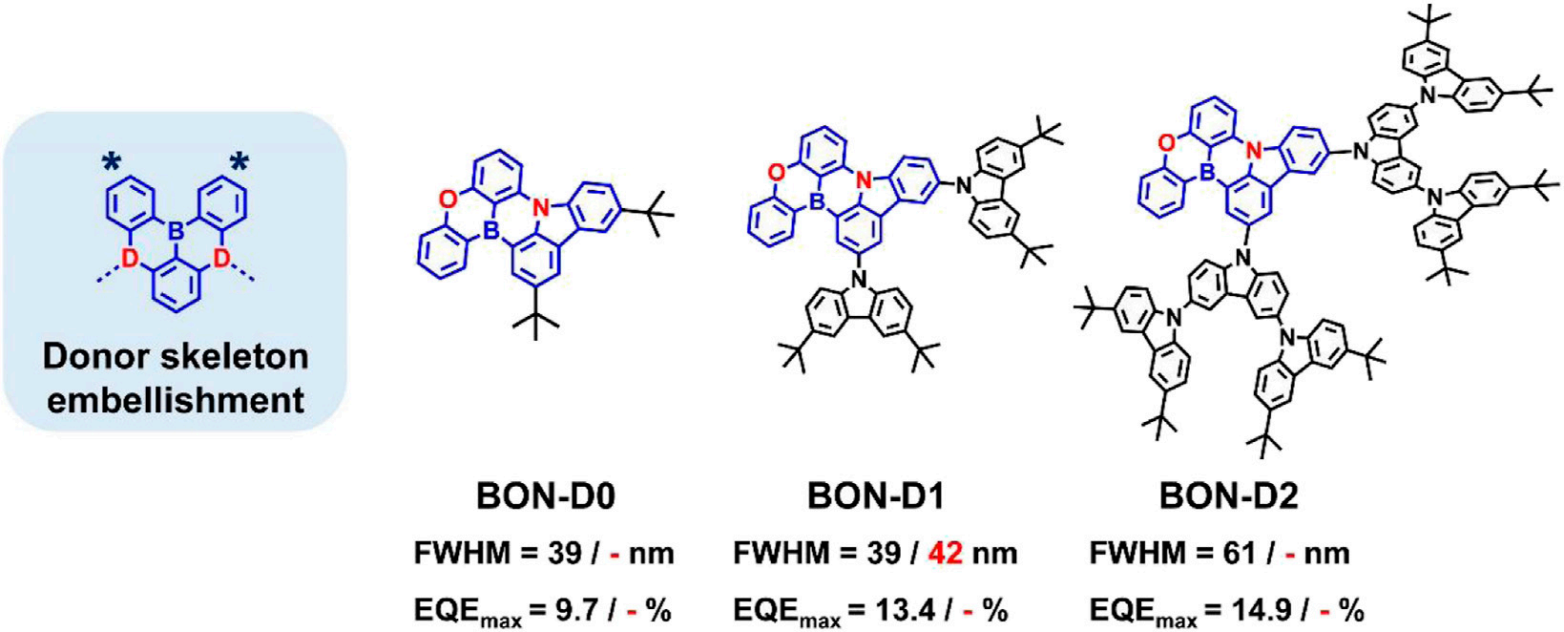
Chemical structures and device performances of donor-skeleton- embellishing MR emitters, where the values represent the FWHMs and EQE_max_s of the OLED devices at low (left) and high (right) doping concentrations, respectively.

The donor wrapping strategy tends to lead to a significant increase in molecular weight and is therefore more suitable for solution-processed devices. For example, Shao et al. designed a series of MR-TADF dendrimers by introducing carbazole dendrons into the periphery of polycyclic aromatic skeleton doped with B, O and N atoms (Liu et al., 2022b) (Figure 5), The target emitters BON-D1 and BON-D2 achieve higher *PLQY*s (≥94%) than the MR core BON-D0 (85%) due to the suppression of intermolecular aggregation by bulk carbazole dendrimers, which partially overcomes unwanted spectral broadening in a wide doping concentration range of 1–30 wt%. When applied to solution-processed OLEDs, the EL emission peak of BON-D1 was only red-shifted from 488 nm to 491 nm with small FWMH increased from 39 nm to 42 nm as the doping concentration increased from 5 wt% to 30 wt%.

## Summary and perspective

As described in this concept, “sterically-wrapped MR fluorophores” is an effective strategy for developing color-tunable, quench-resistant and narrowband emissive materials. Using the newly designed emitters, the corresponding optimized MR-OLEDs can achieve high EQEs of >40% while maintaining extremely small FWHMs of <25 nm over a wide range of concentrations (1–20 wt%). Despite the significant achievements, there are still some challenges and opportunities that should be addressed in the future. On one hand, this strategy is limited to B, N/O-based MR-TADF molecules and how it can be further extended to other systems (e.g., carbonyl/nitrogen- and indole-carbazole-based MR-TADF materials). On the other hand, the efficiency roll-off and device stability issues in wrapped MR-OLEDs remain to be optimized. In this case, one can consider introducing the concepts related to “space-charge-transfer” and “chemical-bonding-enhancement” to increase the *k*_RISC_s and improve the intrinsic stability of the materials. In addition, there is still an urgent need to further develop novel structures, such as the recently reported spatial macrocycles (Fan et al., 2022) and spirofluorene-locked (Liu et al., 2022c) molecules. Given the rapid progress of research in a short period of time, we believe that the above strategy will play an increasingly important role in ultra-wide color gamut OLED displays.

## Data Availability

The original contributions presented in the study are included in the article/Supplementary Material, further inquiries can be directed to the corresponding author.
